# 

**Published:** 1903-01-03

**Authors:** 


					the hospital. "The standard of highest purity."?Lancet. jai*. 3rd, 1903.
CADBURY'S ??, CADBURY'S
cocoa ~1\ jrfl cocoa
ABSOLUTELY PURE. Jgb ?=> NO DRUQS 0E ALKALIES USED.
No. 84f. Vol. XXXIII. [fNtwapapen]
Saturday, Jan. 3rd, 1903.
^Subscript i<m Terms,] pRICH TWOPENCE.

				

